# Spatial and Temporal Progression of Neurodegeneration in Confirmed and Suspected TDP-43 Type C Pathology

**DOI:** 10.1162/IMAG.a.83

**Published:** 2025-07-16

**Authors:** Jane Stocks, Jordan Behn, Elena Barbieri, M-Marsel Mesulam

**Affiliations:** a Department of Psychiatry and Behavioral Sciences, Feinberg School of Medicine, Northwestern University, Chicago, IL, USA 60611; b Mesulam Center for Cognitive Neurology and Alzheimer’s Disease, Feinberg School of Medicine, Northwestern University, Chicago IL, USA 60611; c Department of Physical Medicine and Rehabilitation, Feinberg School of Medicine, Northwestern University, Chicago IL, USA 60611; d Department of Neurology, Feinberg School of Medicine, Northwestern University, Chicago IL, USA 60611

**Keywords:** Neurodegeneration, primary progressive aphasia, TDP-43, atrophy, MRI, longitudinal

## Abstract

The delineation of disease progression in neurodegenerative entities offers neurobiological insights into pathophysiology as well as practical information on natural progression that can be used to gauge the benefits of treatment. In AD, FTLD-tau and FTLD-TDP types A and B such advances have been achieved based on the investigation of presymptomatic mutation carriers. In the absence of dominant mutations, this approach is not feasible in FTLD-TDP type C (TDP-C). Considering the subtlety of the initial symptoms, it is almost certain that the disease had been progressing for years before the first investigations are obtained. We addressed this limitation through an indirect approach based on the fact that neurodegeneration in TDP-C can be asymmetric for 5-6 years after symptom onset. In time, the contralateral hemisphere starts to show the onset of atrophy, the spread of which mirrors the pattern in the affected hemisphere. The unaffected hemisphere therefore offers an opportunity for capturing the very first emergence of atrophy. To that end, we traced the onset and progression of neurodegeneration in TDP-C by analyzing the right hemisphere longitudinally in cases of asymmetric left anterior temporal atrophy. In these cases, TDP-C was either confirmed at autopsy or suspected based on the clinical features and anatomy of atrophy. Structural MRIs were processed using voxel-based morphometry and parcellated into cortical and subcortical regions. W-scores were computed to identify volume loss relative to age-matched controls. Linear mixed-effects models assessed disease progression across regions of interest (ROIs). Results of our analyses reveal that atrophy in TDP-C follows a stereotyped progression within the right hemisphere, beginning in the ventromedial anterior temporal lobe and extending posteriorly and laterally over time. Early atrophy was most prominent in the medial temporal pole (planum polare), perirhinal cortex, entorhinal cortex, and anterior fusiform cortex, with subcortical involvement initially limited to the amygdala. Voxelwise and ROI-based analyses confirmed that cortical atrophy preceded and exceeded amygdala atrophy in most cases, suggesting greater neocortical vulnerability. Longitudinal linear mixed-effects models identified the greatest volume loss in medial temporal ROIs and the amygdala, following a consistent anterior-to-posterior gradient over time. These findings reconstruct the spatial and temporal progression of TDP-C pathology using the initially unaffected hemisphere as a proxy for early disease stages. The stereotyped trajectory of atrophy aligns with neuropathological patterns and offers critical insights into disease progression, aiding therapeutic evaluation.

## 1. Introduction

Frontotemporal lobar degeneration (FTLD) is an umbrella term for a group of neurodegenerative diseases characterized by the accumulation of toxic protein aggregates in the central nervous system. Clinically, FTLD is highly heterogeneous, with its canonical syndromes presenting as distinct combinations of progressive deficits in movement, behavior, language, and/or cognition. Approximately half of FTLD cases are attributed to transactive response DNA binding protein 43 kDa (TDP-43) pathology (Dickson et al., 2011), which is further divided into subtypes based on the patterns of neuronal intranuclear inclusions, neuronal cytoplasmic inclusions and dystrophic neurites (Lee et al., 2017; Mackenzie et al., 2011). Among these subtypes, FTLD-TDP Type C (TDP-C) is notable for the abundance of long dystrophic neurites predominantly in the superficial neocortex and cytoplasmic inclusions in subcortical regions (Mackenzie et al., 2011).

TDP-C is distinct among neurodegenerative diseases due to its unique and consistent predilection for the anterior portions of the temporal lobe (ATL). The neurodegeneration of TDP-C, detected as atrophy in MRI, is frequently asymmetrical. The clinical manifestations of left predominant disease include severe naming impairments and word recognition deficits, a combination known as the semantic variant of Primary Progressive Aphasia (PPA) (Borghesani et al., 2020; Mesulam et al., 2021; Mesulam et al., 2022; Mesulam et al., 2023; Rohrer et al., 2010). Right-predominant disease, on the other hand, is associated with associative agnosias (i.e., non-verbal recognition impairments), socioemotional dysfunction, and behavioral abnormalities (Gefen et al., 2013; Younes et al., 2022).

The identification of presymptomatic mutation carriers in several neurodegenerative diseases such as Alzheimer’s, FTLD-tau, and FTLD-TDP types A and B have led to fundamental data on the very initial stages of disease. TDP-C is arguably the only major neurodegenerative disease without underlying disease-causing mutations or familial associations. It is therefore necessary to find other approaches for tracing the onset and early progression of neurodegeneration. This information is critical for clarifying the initial target of the disease and for constructing a temporal trajectory of progression that can be used to gauge treatment effects.

Based on observations on 32 autopsy verified TDP-C cases with left predominant disease, we had found that neurodegeneration emerges in the right hemisphere as well but generally 5-6 years after symptom onset (Mesulam et al., 2023). The contralateral onset and spread of atrophy mirrors the pattern in the affected hemisphere. The unaffected hemisphere therefore offers an opportunity for capturing the very first stages of atrophy and its subsequent expansion. Here, we used quantitative MRI morphometry to image the initially intact or minimally affected right ATL and its subsequent progressive atrophy as a surrogate for the initial trajectory of neurodegeneration caused by TDP-C. Our cohort consisted of individuals with asymmetric left anterior temporal atrophy and the clinical syndrome of svPPA. The atrophy patterns in the right ATL were quantified at three time points, enabling the reconstruction of the progression from stage zero. By doing so, we provide critical insights into the progression trajectory of TDP-C pathology.

## 2. Methods

### 2.1. Participants

Forty right-handed participants with PPA due to TDP-C were included in the analyses. Participants were recruited from our PPA Research Program, a large longitudinal study that is funded by the National Institute on Aging. Participants were evaluated at the Mesulam Cognitive Neurology and Alzheimer’s Disease Center of Northwestern University Feinberg School of Medicine in Chicago, IL. The study was approved by the Northwestern University Institutional Review Board and conducted in compliance with the Declaration of Helsinki. All participants provided written informed consent prior to entering the study. TDP-C was confirmed at autopsy in 18 participants through neuropathological examination, which identified hallmark features, including immunoreactive TDP-43 short and long dystrophic neurites, neuronal cytoplasmic inclusions, neuronal loss and gliosis and the absence of neuronal intranuclear inclusions (Lee et al., 2017). For an additional 22 participants, the diagnosis was based on clinical and imaging criteria, including prominent word comprehension impairment and asymmetric left-sided anterior temporal lobe atrophy, consistent with the patterns observed in TDP-C cases. Two subjects who carried a diagnosis of semantic dementia and demonstrated significant right-ward asymmetry of atrophy at initial visit were excluded from the present analyses. Forty structural MRI scans were available for subjects at Visit A (baseline), 28 scans at Visit B (2-year follow-up), and 13 scans at Visit C (4-year follow-up). Healthy control participants were defined by intact performance on standardized neuropsychological testing and the absence of reported or observed cognitive decline, based on clinical consensus and longitudinal follow-up.

### 2.2. MRI Acquisition and Preprocessing

Structural T1-weighted 3D images were acquired using MP-RAGE sequences on a Siemens TIM 3T Trio or Prisma scanner (slice thickness: 1 mm, TR = 2300 ms, TE = 2.91 ms, TI = 900 ms, flip angle = 9°, FOV = 256 × 256mm). Images were manually re-oriented to the AC-PC line using SPM12 (https://www.fil.ion.ucl.ac.uk/spm/software/spm12/) and then pre-processed using the Computational Anatomy Toolbox 12 toolbox (CAT12, Gaser et al. 2022; https://neuro-jena.github.io/cat/) using Matlab 2024a. Image pre-processing included initial denoising, correction for intensity bias, and affine registration, utilizing the unified segmentation approach from SPM12 (Ashburner & Friston, 2005). Further voxel-based processing includes skull stripping, local intensity normalization, adaptive maximum a posteriori (AMAP) segmentation, co-registration, and normalization to a standardized reference space using Geodesic Shooting. The gray matter volume images, after modulation and normalization to a 1.5 × 1.5 × 1.5 mm resolution, were smoothed with an 8 mm Gaussian kernel. Detailed descriptions of the pre-processing pipeline can be found in the CAT12 Manual (https://neuro-jena.github.io/cat12-help/). Quality control of the GM volume images was conducted using CAT12’s image quality rating (IQR), a composite measure incorporating noise contrast, inhomogeneity contrast, and resolution quality.

Following the initial pre-processing steps, brain images were parcellated into distinct regions of interest (ROIs) for both cortical and subcortical structures. Cortical parcellation was performed using the Glasser et al. (2016) multimodal parcellation atlas, which provides high-resolution delineation of cortical areas based on structural and functional imaging features. Subcortical regions were segmented using the Tian et al. (2020) functional connectivity-based subcortical atlas.. All subsequent analyses were performed and visualized only on the right hemisphere.

### 2.3. W-Score Calculations

W-scores are standardized residual scores used to quantify regional or voxelwise volume loss while accounting for normative effects of interest and have been widely used in neuroimaging paradigms (La Joie et al., 2012). First, a general linear model (GLM) was applied to the healthy control group at baseline (i.e., Visit A) to model the relationship between age and regional brain volume. Predicted values were derived from the GLM, and W-scores were computed by subtracting the predicted values from the observed values and standardizing the differences using the residual standard deviation from the control group. W-scores were first calculated for region-of-interest (ROI) data based on the Glasser HCP atlas for cortical regions and the Tian subcortical atlas for subcortical structures. These ROI-level W-scores provided standardized metrics of deviation from expected brain volume in healthy age-matched participants. In the ROI analyses, the percentage of TDP-C subjects with W-scores below −1.5 in each region was visualized using the ggseg package in R (Mowinckel & Vidal-Piñeiro, 2020).

The same approach was then applied to voxelwise data to achieve higher spatial resolution. This voxelwise analysis allowed for the generation of detailed maps highlighting localized deviations in brain volume, offering a more granular perspective on patterns of atrophy across the brain. A threshold of W-scores under −1.5 was used to identify regions of abnormal atrophy, again representing volumes more than 1.5 standard deviations below the expected range in the control group. Regions meeting this threshold were considered to exhibit significant volume loss. Visualization of atrophy was performed at all visits at the 25% threshold, and only in visit A at an 80% threshold, to assess the precise location of the earliest site of atrophy. Percentages reflect the proportion of participants that met the −1.5 W-score threshold at each voxel.

To further characterize the pattern of atrophy in individual subjects, W-score maps were generated for each participant. A qualitative assessment was then performed to evaluate the presence or absence of cortical anterior temporal atrophy (i.e., anterior to the limen insulae) and amygdala atrophy. Given our interest in identifying the relative prominence of cortical versus subcortical involvement in early disease, we applied a forced-ranking approach to directly compare atrophy severity between these two regions within each subject. Raters reviewed the W-score maps to determine if anterior temporal cortex or amygdala exhibited more visually apparent atrophy. Subjects were then classified as showing either greater cortical atrophy, greater amygdala atrophy, or an equivocal pattern in cases where atrophy appeared minimal or equally distributed across regions. This approach allowed us to assess whether early cortical involvement was a consistent individual-level feature of TDP-C, complementing the group-level findings from ROI- and voxel-based analyses.

### 2.4. Statistical Analyses

To investigate the relationship between disease duration and brain volume decline, linear mixed-effects (LME) models were employed using the nlme package in R (Pinheiro et al., 2017). This statistical approach was chosen for its ability to account for both fixed and random effects, allowing for robust modeling of repeated measures data while accounting for individual variability. The analysis focused on brain volume within the predefined regions of interest (ROIs) delineated using the Glasser HCP (2016) and Tian (2020) subcortical atlases. The fixed-effects formula specified the relationship between brain volume and disease duration, operationalized as years since symptom onset (rather than time since the first scan), to model change relative to disease progression. This approach accounts for the fact that participants entered the study at different stages of disease and enables a common time axis anchored to symptom onset. Random effects were included to account for inter-individual variability, with random intercepts specified for each participant. The use of a random intercept structure allowed the model to capture baseline differences in brain volume across individuals while maintaining a consistent slope for the effect of disease duration. The output of the LME models included fixed-effect coefficients, representing the average effect of disease duration on brain volume, and random-effect estimates, reflecting participant-specific deviations from the average trajectory. The average slope representing the effect of disease duration on brain volume was visualized for each region. Cortical ROIs were mapped using the ggseg package in R (Mowinckel & Vidal-Piñeiro, 2020), based on the Glasser HCP atlas (Glasser et al., 2016), to highlight spatial patterns of cortical atrophy. Subcortical regions were visualized with orthogonal slice views using the ortho2 function in the neurobase package. Only regions surpassing a stringent FDR-corrected significance threshold were included in the visualization to emphasize the most robust effects.

## 3. Results

### 3.1. Participants

Full participant demographics are reported in Table 1. Control (CON) group included 64 participants only at Visit A. The mean age at Visit A was similar between groups, with 63.0 years (SD = 6.5) for the TDP-C group and 63.5 years (SD = 6.5) for the CON group. The percentage of female participants was slightly lower in the TDP-C group (47.6%) compared to the CON group (55.2%). Both groups were predominantly White, non-Hispanic, with 96% in the TDP-C group and 88% in the CON group. The TDP-C group had a mean Clinical Dementia Rating (CDR) Global score of 0.42 (SD = 0.3) at the initial visit, reflecting mild overall clinical impairment, whereas the CON group had a score of 0.0, indicating no cognitive impairment. The mean age of symptom onset in the TDP-C group was 58.5 years (SD = 1.8).

### 3.2. Regional Atrophy Pattern in ROIs

**Figure 1.**
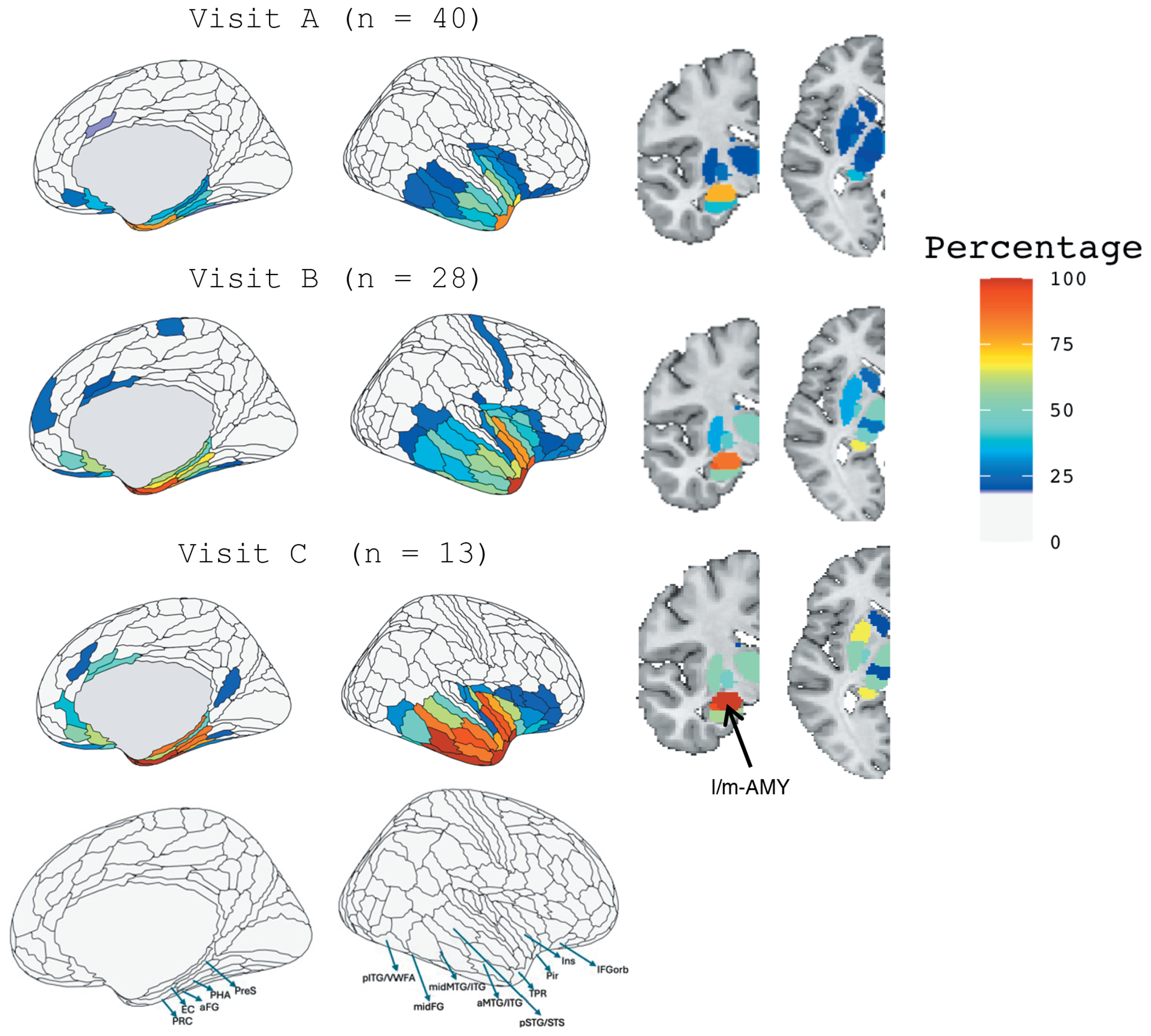
Percentage of Participants with Brain Volume W-Scores Below −1.5 in Right Hemisphere Regions of Interest (ROIs) at Visits A, B, and C This figure illustrates the percentage of participants with brain volume W-scores below −1.5, based on comparison to normal controls, in 180 right hemisphere ROIs derived from the Glasser et al. (2016) HCP-MMP atlas for visits A, B, and C. Subcortical regions are parcellated according to the Tian et al. (2020) atlas; labels are intended to aid interpretability and do not reflect definitive subregional boundaries. Visualizations are thresholded at 25%, displaying ROIs where 25% or more participants fall below the W-score threshold across the three visits. PRC = perirhinal cortex, EC = entorhinal cortex, aFG = anterior fusiform gyrus, PHA = parahippocampal area, PreS = presubiculum, pTG/VWFA = posterior temporal gyrus/visual word form area, midFG = middle fusiform gyrus, midMTG/ITG = middle and inferior temporal gyri, aMTG/ITG = anterior middle and inferior temporal gyri, pSTG/STS = posterior superior temporal gyrus and superior temporal sulcus, TPR = temporal pole region, Pir = piriform cortex, Ins = insula, and IFGorb = orbital portion of the inferior frontal gyrus. l/m-AMY = lateral and medial amygdala

Figure 1 illustrates the percentage of participants with W-scores < −1.5 in the ROI analysis of the right hemisphere. Visualizations are thresholded at 25%, highlighting ROIs where at least 25% of participants exhibit significant atrophy across the three visits. At Visit A, regions with the highest proportion of participants (>80%) exhibiting atrophy were the temporal polar cortex (HCP-MMP regions “TGd” and “TGv”) and perirhinal cortex (HCP-MMP region PeEc). Subcortically, the lateral and medial subdivisions of the amygdala were most affected, in roughly 70% of subjects, although to a slightly lower extent than cortical regions. By Visit B, nearly all participants (close to 100%) demonstrated atrophy in the regions identified at Visit A. Additional areas of significant atrophy emerged, including insular cortex (HCP-MMP regions posterior insula (PoI1) and parainsula (Pi)), as well as the the piriform cortex. Subcortically, the amygdala (lateral and medial subdivisions) remained the primary site of volume loss. At Visit C, atrophy expanded further, encompassing posterior lateral and medial regions of the temporal lobe. These included the anterior and middle portions of the middle and inferior temporal gyri, as well as parahippocampal and entorhinal cortices and pre-subiculum. Subcortical involvement also became more extensive, with 50-70% of participants demonstrating volume loss in the nucleus accumbens, anterior putamen, and both the anterior and posterior divisions of the hippocampus. A separate analysis of autopsy-confirmed and clinically suspected TDP-C cases is presented in Supplemental Figure 1; both subgroups showed similar spatial patterns of atrophy, with the autopsy-confirmed group exhibiting slightly greater atrophy overall.

### 3.3. Voxelwise Atrophy Pattern

**Figure 2.**
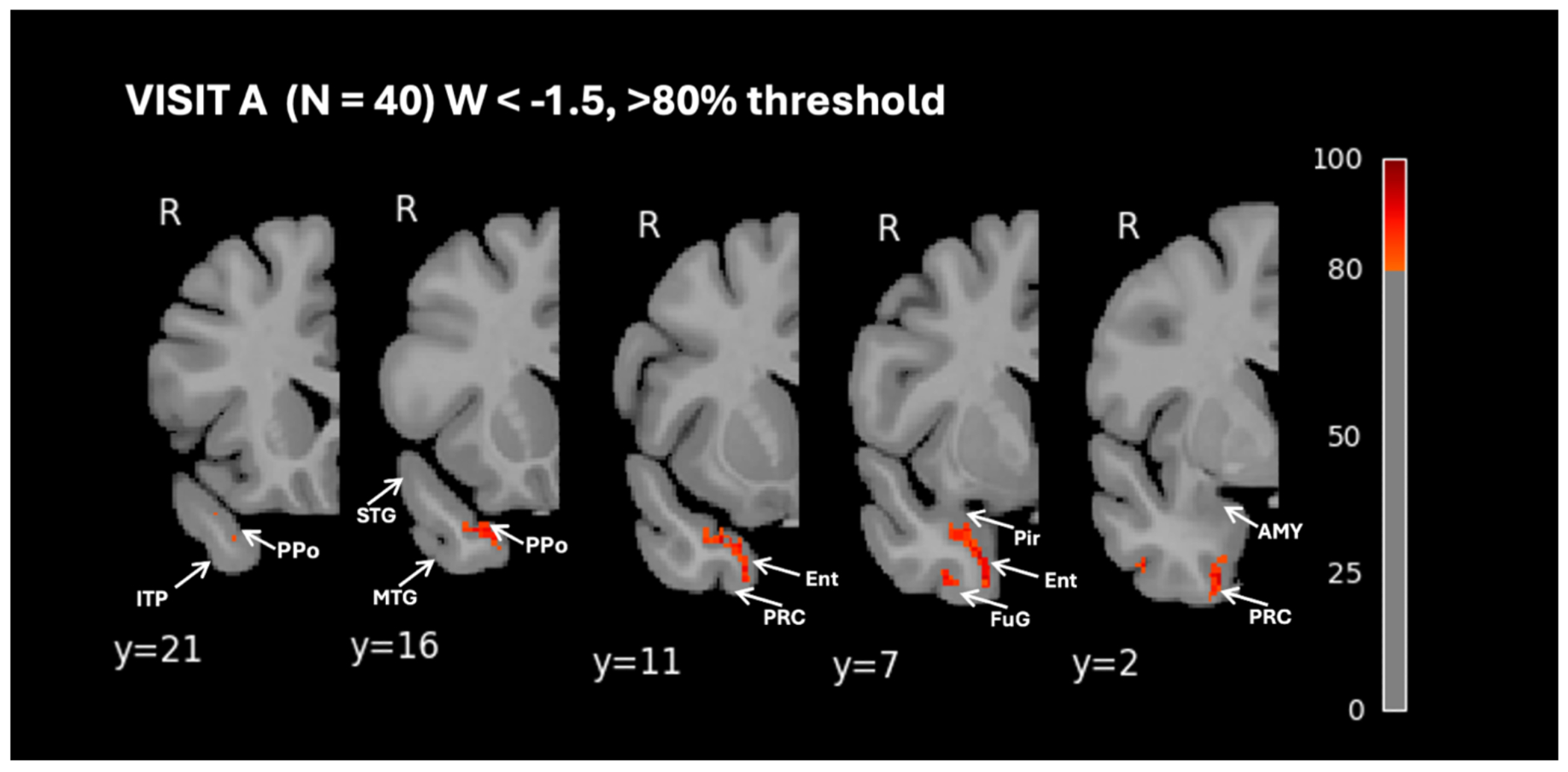
High Threshold Percentage of Participants with Brain Volume W-Scores Below −1.5 Visualized Voxelwise in the Right Hemisphere at Visit A This figure presents voxelwise visualizations of the percentage of participants with brain volume W-scores below −1.5, in the right hemisphere, for visit A only. Data are thresholded at 80%, with axial slices showing areas where 80% or more participants fall below the W-score threshold. Figure labels based on Mai et al. (2015); labels are intended to aid interpretability and do not reflect definitive subregional boundaries. Ppo = Planum polare, ITP = Inferior temporopolar region, STG = Superior temporal gyrus, MTG = Middle temporal gyrus, Pir = Piriform Cortex, Ent = Entorhinal cortex, PRC = perirhinal cortex, AMY = amygdala, FuG = fusiform gyrus

**Figure 3.**
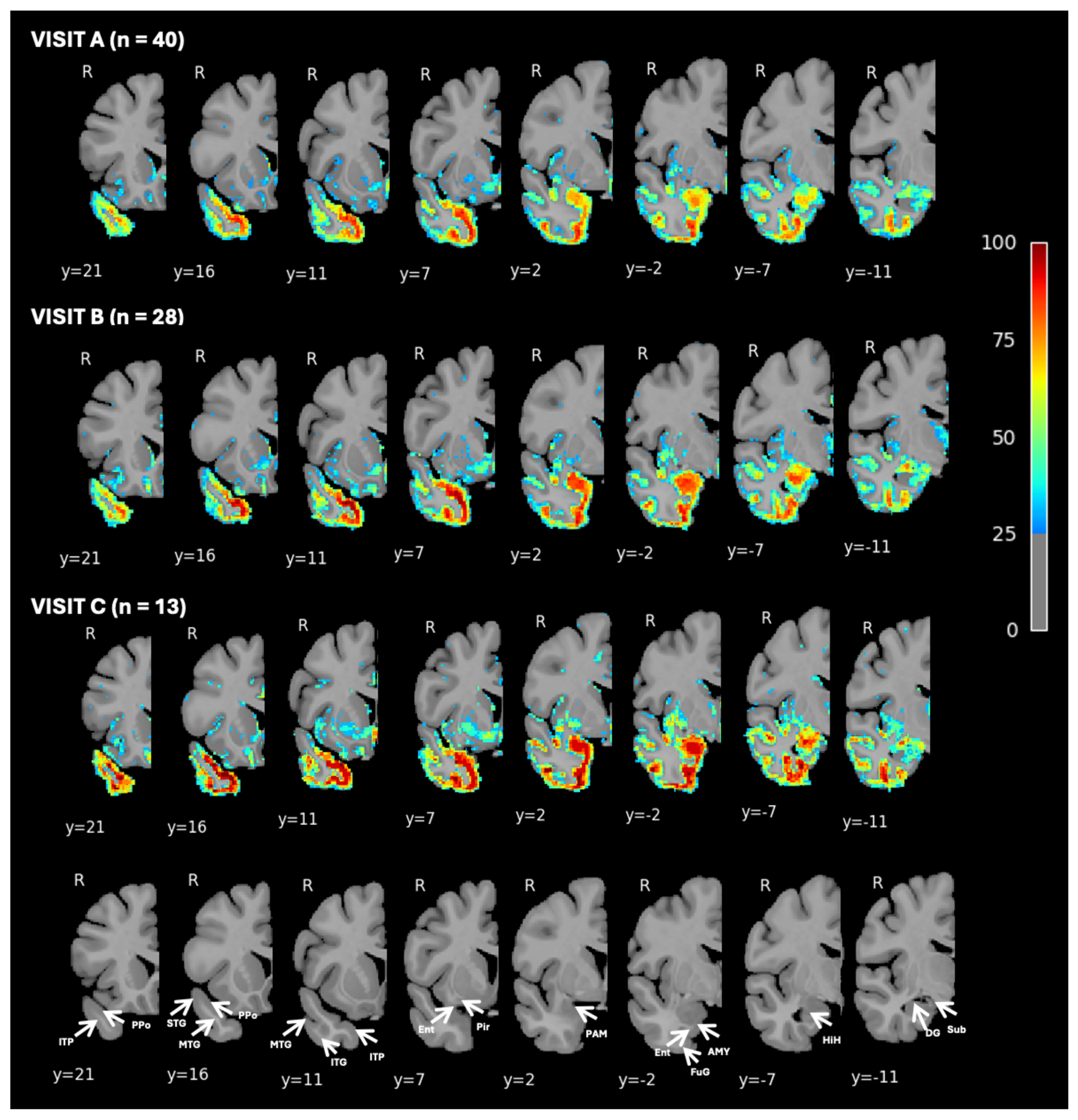
Percentage of Participants with Brain Volume W-Scores Below −1.5 Visualized Voxelwise in the Right Hemisphere at Visits A, B, and C This figure presents voxelwise visualizations of the percentage of participants with brain volume W-scores below −1.5, compared to normal controls, in the right hemisphere, for visits A, B, and C. Data are thresholded at 25%, with axial slices showing areas where 25% or more participants fall below the W-score threshold. The voxelwise analysis provides detailed insight into specific regions of interest across the right hemisphere. Figure labels based on Mai et al. (2015); labels are intended to aid interpretability and do not reflect definitive subregional boundaries. Ppo = Planum polare, ITP = Inferior temporopolar region, STG = Superior temporal gyrus, MTG = Middle temporal gyrus, ITG = Inferior temporal gyrus, Pir = Piriform Cortex, Ent = Entorhinal cortex, PAM = periamygdaloid complex, AMY = amygdala, FuG = fusiform gyrus, HIH = hippocampal head, DG = dentate gyrus.

To investigate the progression of right-hemisphere atrophy in greater detail, a voxelwise analysis was conducted, providing higher spatial resolution and precise localization of cortical and subcortical volume loss. Figure 2 presents the percentage of participants with W-scores < −1.5 at Visit A only, thresholded at 80% to highlight voxels where at least 80% of participants exhibit significant atrophy. These voxels represent the regions in which nearly all subjects show some degree of atrophy, thus serving as the hypothesized initial site of atrophy. This included regions such as the planum polare, the anterior entorhinal cortex, perirhinal cortex, and anterior fusiform gyrus. Notably, regions of the amygdala and adjacent peri-amygdaloid and piriform cortex, did not meet this threshold. To better capture the spatial and temporal progression of atrophy, we visualize voxelwise atrophy of the right hemisphere across 3 longitudinal study visits. Figure 3 presents the findings from voxelwise percentage of participants with W-scores < −1.5 at all visits, thresholded at 25% to highlight voxels where at least 25% of participants exhibit significant atrophy. As described above, atrophy at Visit A is greatest in ventromedial portions of the temporal pole, anterior to the limen insulae. A lesser, but notable, degree of atrophy was also observed in the periamygdaloid and piriform cortices, with a lower proportion of participants (~70%) showing significant atrophy in the amygdala proper. By Visits B and C, the regions most affected by atrophy remained largely consistent with those identified at Visit A. However, nearly all participants at follow-up visit displayed significant subcortical atrophy, particularly in the amygdala and parahippocampal gyrus. Between Visits A and B, the progression of atrophy extended caudally along the ventral surface of the anterior temporal lobe, encompassing additional regions of the fusiform and inferotemporal cortices. Subgroup analyses are presented in Supplemental Figures 2–4. The voxelwise atrophy pattern at the 80% threshold (Supplemental Figure 2) and the 25% threshold across all visits (Supplemental Figures 3–4) was consistent between autopsy-confirmed and clinically suspected cases, with slightly more extensive atrophy observed in the autopsy group.

To further assess the differential atrophy of cortical temporal pole (i.e., anterior to the limen insulae) and subcortical (i.e., amygdala) atrophy, we performed a qualitative rating of single subject atrophy maps. While group-level analyses suggested early cortical involvement, this additional step was conducted to evaluate whether the predominance of cortical atrophy held true at the individual level. Qualitative ratings revealed that cortical anterior temporal atrophy was clearly present in 32 out of 40 cases (80%), while amygdala atrophy was evident in 20 out of 40 cases (50%). Forced-ranking of atrophy severity indicated that cortical atrophy was greater than amygdala atrophy in 29 of 40 cases (73%). Ten cases were classified as equivocal, typically due to minimal atrophy in both regions, or in some instances, substantial atrophy in both, making direct ranking difficult. When excluding these equivocal cases, cortical atrophy was identified in 29 of 30 cases (97%), while amygdala atrophy was present in 17 of 30 cases (57%). Forced-ranking within this subset further supported a predominance of cortical atrophy, with 23 of 30 cases (77%) showing greater cortical than amygdala involvement. These findings suggest a consistent pattern of early cortical atrophy in TDP-C.

### 3.4. Rates of Volume Decline

**Figure 4.**
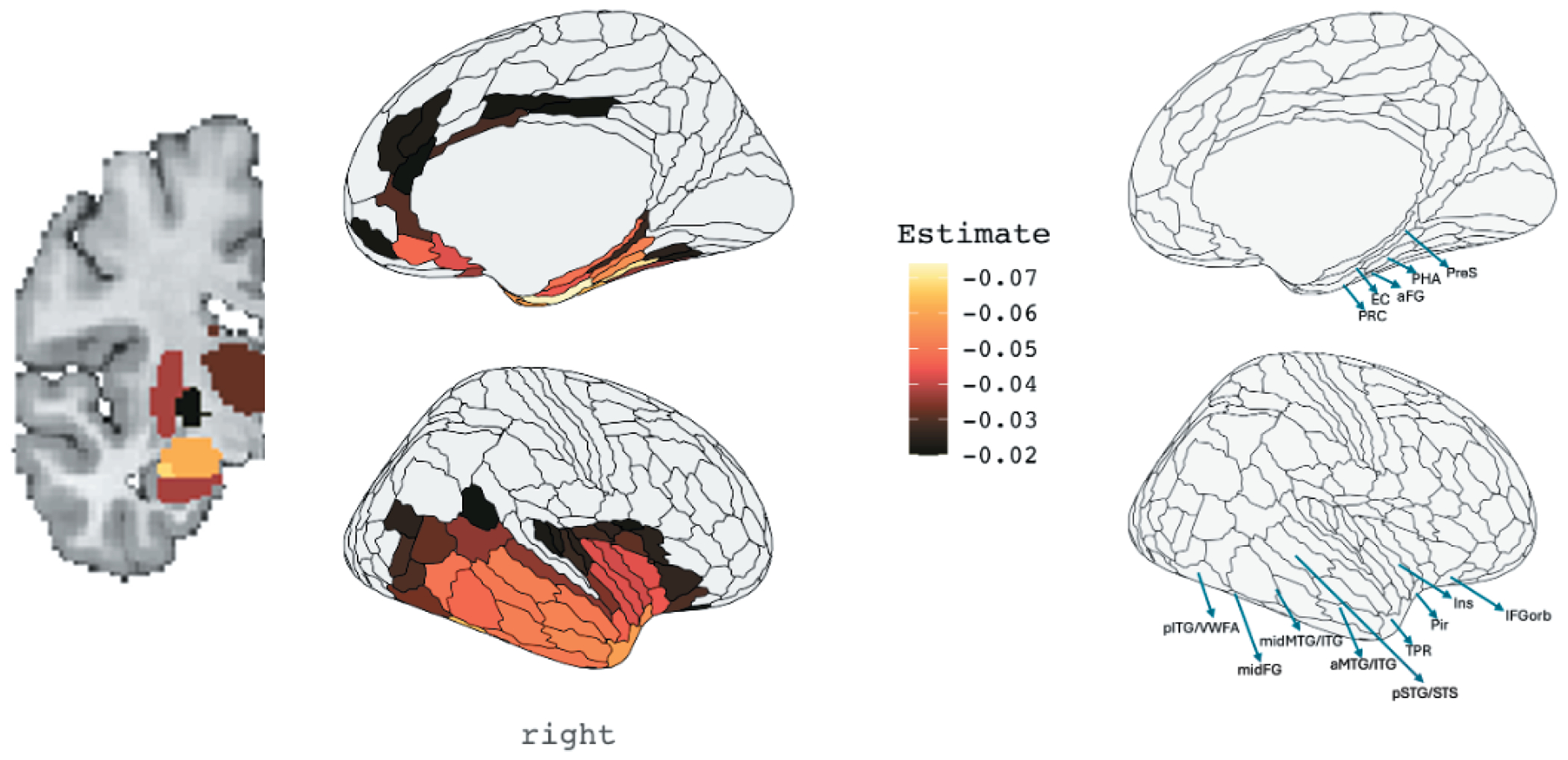
Effects of Years Since Symptom Onset on Brain Volume This figure displays the results of linear mixed-effects models examining the effect of disease duration on brain volume across 180 right-hemisphere regions of interest (ROIs) from the Glasser et al. (2016) HCP-MMP atlas and 16 subcortical ROIs from Tian et al. (2020); labels are intended to aid interpretability and do not reflect definitive subregional boundaries. Lighter yellow values correspond to faster rates of volume loss per year since symptom onset. The colorbar is thresholded to display only ROIs exceeding a stringent FDR-corrected significance level. PRC = perirhinal cortex, EC = entorhinal cortex, aFG = anterior fusiform gyrus, PHA = parahippocampal area, PreS = presubiculum, pTG/VWFA = posterior temporal gyrus/visual word form area, midFG = middle fusiform gyrus, midMTG/ITG = middle and inferior temporal gyri, aMTG/ITG = anterior middle and inferior temporal gyri, pSTG/STS = posterior superior temporal gyrus and superior temporal sulcus, TPR = temporal pole region, Pir = piriform cortex, Ins = insula, and IFGorb = orbital portion of the inferior frontal gyrus.

To better understand which regions exhibited progressive volume loss over time, we examined the statistical relationship between W-scores and years since symptom onset using linear mixed-effects models. This approach accounted for variability in disease duration among participants, enabling identification of ROIs with significant atrophy progression as a function of disease duration. Figure 4 illustrates the coefficient estimates from these models, representing the rate of volume decline (slope) per year since symptom onset for each ROI. The regions with the steepest negative slopes, indicating the greatest rates of atrophy over time, included the perirhinal cortex, temporal polar cortex, the lateral and medial subdivisions of the amygdala, and the uncus/parahippocampal cortex. Other areas of the temporal lobe also showed significant volume loss, particularly in the lateral and ventromedial temporal cortices. Notably, the decline followed an anterior-to-posterior gradient within the temporal lobe, with more posterior regions showing slower rate of atrophy over time. Subgroup analyses of atrophy rates by diagnostic confirmation are shown in Supplemental Figure 5. Both autopsy-confirmed and clinically suspected cases demonstrated similar regional patterns and anterior-to-posterior gradients of volume decline. Longitudinal atrophy in the autopsy-confirmed group was more posteriorly distributed within the temporal lobe, possibly reflecting greater baseline atrophy at the temporal pole. In contrast, the clinically suspected group showed more diffuse subcortical involvement over time, with longitudinal progression extending beyond the amygdala to include the hippocampus and basal ganglia.

## 4. Discussion

This study aimed to delineate the progression of neurodegeneration in FTLD-TDP Type C by leveraging the asymmetric nature of its atrophy pattern. Given that neurodegeneration in TDP-C can remain unilateral, most commonly in the left hemisphere, for several years after symptom onset, we used the right hemisphere as a historical surrogate to infer the earliest stages of disease progression. This novel approach is rare in that it is based on a longitudinal cohort of individuals with autopsy-confirmed or suspected TDP-C pathology, providing a unique opportunity to examine the natural history of this disease in the absence of presymptomatic genetic models. To that end, we conducted ROI and voxelwise analyses of right hemisphere atrophy using a W-score approach in 40 subjects with suspected or autopsy-confirmed TDP-C. Data were available for subjects in up to 3 study visits, enabling the study of longitudinal patterns of spatial progression of atrophy.

In ROI-wise analyses, we identified the temporal pole and anteromedial subdivisions of the right temporal lobe as the regions in which the highest proportions of subjects demonstrate volume loss. Voxelwise analyses enabled a more precise localization of the initial site of volume loss, which was noted to be the ventromedial circumpolar region, anterior to the limen insulae, encompassing perirhinal, entorhinal, and fusiform cortex. Longitudinal progression of atrophy at subsequent visits extended caudally along the ventral surface of the anterior temporal lobe as laterally, toward the inferotemporal and middle temporal gyri, and medially, to encompass subcortical regions of the amygdala, uncus, and hippocampus. Qualitative assessment of individual subject atrophy maps revealed that cortical anterior temporal atrophy was more prevalent than amygdala atrophy, with 80% of cases showing clear cortical involvement compared to 50% with amygdala atrophy. Finally, linear mixed-effects analyses on the rate of atrophy as a function of disease duration in subjects revealed right hemisphere temporopolar cortex and medial temporal regions showing the greatest rate of volume loss over time, with an overall anterior-to-posterior gradient of effect. Findings were consistent across autopsy-confirmed and clinically suspected subgroups (Supplemental Figures 1–5), with slightly greater overall atrophy observed in the autopsy-confirmed group. Minor differences emerged only in the longitudinal analyses, where the autopsy-confirmed group showed more posterior extension of atrophy, possibly due to greater baseline involvement of anterior regions.

Our findings are novel in that, to our knowledge, no study to date has examined longitudinal anterior temporal atrophy at the voxelwise level in the right hemisphere. Asymmetric anterior temporal lobe atrophy in autopsy-confirmed cases of TDP-C pathology (Mesulam et al., 2022; Rohrer et al., 2010; Rohrer et al., 2009) and in semantic variant PPA (Rogalski et al., 2011) has been well documented; with atrophy either absent in the right hemisphere or constrained to anteriormost portions of the temporal pole. In Bocchetta et al. (2020), a 4-stage model of atrophy progression was proposed, with atrophy at the initial stage involving the left amygdala and medial, lateral and polar temporal cortex. Stage 1 also included the right medial temporal lobe, noting this region is the first to be implicated in the contralateral hemisphere. In another study, Borghesani et al (2020) analyzed autopsy-confirmed TDP-C cases and observed that, regardless of whether atrophy was left- or right-lateralized, the progression of volume loss followed a medial-to-lateral gradient. Their analysis was based on a four-part parcellation of the temporal lobe, which they developed for their study. Among these subdivisions, the inferior-medial region, which encompassed Brodmann areas (BA) 35 and 36, exhibited the most significant atrophy, further emphasizing the early involvement of perirhinal and adjacent ventromedial temporal structures in TDP-C pathology. Alternative methods of estimating the site of peak atrophy have been proposed, including using and individualized “epicenter” approach, in which seed-based functionally connectivity patterns are compared to see which best recapitulates a subject’s atrophy pattern (Seeley et al., 2009). Using this approach, Brown et al. (2019) identified the left ventromedial temporal pole as the disease epicenter in the greatest proportion of svPPA subjects, with additional epicenters in the right ventromedial temporal pole, left ventral pole/inferior temporal cortex, and left ventral temporal pole/ fusiform.

Our findings align with prior staging models of TDP-C atrophy that emphasize early involvement of medial and ventral anterior temporal structures. For instance, Bocchetta et al. (2020) proposed that the right medial temporal lobe is among the first regions affected in the contralateral hemisphere, and Borghesani et al. (2020) identified the inferior-medial temporal region—encompassing Brodmann areas 35 and 36 (perirhinal and adjacent cortex)—as the most atrophied in autopsy-confirmed TDP-C cases. While these studies used different parcellation schemes, our voxelwise analysis similarly identifies the ventromedial anterior temporal cortex—including the perirhinal, entorhinal, and anterior fusiform regions—as the earliest site of atrophy.

Our results indicate that the initial site of atrophy in TDP-C is the ventromedial portion of the anterior temporal lobe, particularly the medial temporal pole (i.e., planum polare), perirhinal, entorhinal, and anterior fusiform cortices. These regions form a highly interconnected network within the ventromedial temporal lobe, characterized by dense reciprocal projections with limbic and neocortical structures. Neuroanatomical, functional, and structural studies of the subdivisions of the temporal pole have gained increasing attention in recent years, highlighting its distinct cytoarchitectonic organization and connectivity patterns (Ding et al., 2009). Dorsal temporal pole, closely linked to the superior temporal gyrus, is primarily involved in auditory and language-related processing, whereas the ventral temporal pole has extensive connections with the inferior temporal and fusiform cortices, supporting high-level visual and semantic integration (Pascual et al., 2015; Persichetti et al., 2021). Meanwhile, the medial paralimbic portions (BA 35-36) are more strongly connected to limbic regions such as the amygdala and orbitofrontal cortex, playing a key role in emotional and salience processing (Olson et al., 2007). Anterior to the limen insulae, medial temporopolar cortex becomes the planum polare, with the ventral portion of planum polare appearing to be continuous with perirhinal cortex (Mesulam, 2023). These findings underscore the temporal pole’s role as a multimodal hub, integrating sensory, cognitive, and affective information across distributed neural networks. The selective vulnerability of the ventromedial anterior temporal cortex in TDP-C may relate to its dense connectivity and integrative function—features shared by transmodal hubs that have been proposed as susceptible to neurodegeneration due to factors such as high metabolic demand, long-range connectivity, and prolonged postnatal development (Barbas, 2015; Buckner et al., 2009; Seeley et al., 2009). Additionally, the presence of large, projection-rich pyramidal neurons in these paralimbic areas may increase their susceptibility to TDP-43 pathology, consistent with emerging models of cell- and layer-specific vulnerability (Mesulam, 2023; Ohm et al., 2022).

The dominant (typically left) hemisphere preponderance of atrophy in TDP-C and svPPA has been well established (Mesulam et al., 2022; Rohrer et al., 2010; Rohrer et al., 2009), yet asymmetric atrophy of the nondominant (usually right) hemisphere has also been documented in a subset of cases (Borghesani et al., 2020). When atrophy is right-lateralized, patients more commonly present with either a behavioral variant FTD syndrome (Thompson et al., 2003), or with associative agnosia, including deficits in face and object recognition, leading to a clinical diagnosis of semantic dementia (Kumfor et al., 2016; Neary et al., 1998). Early in disease, truly equal bilateral atrophy is uncommon, and an irregular distribution in which some regions exhibit greater atrophy in the left hemisphere while others are more affected on the right is even more unusual. The pattern of longitudinal atrophy observed in the present study—where early right hemisphere atrophy is confined to a specific portion of the temporal lobe before extending posteriorly along the ventral and inferior portions of the temporal lobe as well as into limbic regions—is consistent with prior literature on TDP-C atrophy progression (Mesulam et al., 2022; Rogalski et al., 2011). The fact that right hemisphere temporal pole atrophy mirrors what is typically observed in the left aligns with evidence suggesting that the two hemispheres are well-mirrored and function as an integrated semantic network (Bonner & Price, 2013; Herlin et al., 2021; Pascual et al., 2015). Object naming and word comprehension deficits result from damage to language-dominant (typically left) temporopolar regions (Gefen et al., 2013), whereas behavioral control, person identification, and nonverbal object recognition depend more critically on the right hemisphere (Franzen & Myers, 1973; Hurley et al., 2018; Olson et al., 2013). Additionally, the left temporal pole has been shown to maintain stronger connections with language-related regions than in the right hemisphere, including the inferior frontal gyrus (e.g., Broca’s area) and the temporoparietal junction (e.g., Wernicke’s area) (Hurley et al., 2015), further distinguishing its role in linguistic processing from that of the right hemisphere.

Although the clinical diagnosis of svPPA with characteristic left anterior temporal atrophy is strongly predictive of TDP-C, the absence of pathological confirmation in a subset of participants is noted to be a limitation. To address this, we performed all primary analyses separately in autopsy-confirmed and clinically suspected subgroups. As detailed in the supplemental figures, the spatial patterns of atrophy were highly consistent across groups, with only subtle differences observed in longitudinal progression. These subgroup findings support the robustness and generalizability of the main results. Another limitation is the relatively coarse temporal resolution, with study visits occurring approximately every two years. This may obscure finer-grained progression dynamics, particularly in early disease stages. Participant attrition over time (40 at baseline, 28 at the second visit, and 13 at the third) may also bias later time points toward individuals with slower progression or greater study retention. However, linear mixed-effects models help address this by modeling individual trajectories and using all available data. Additionally, while our approach assumes that atrophy in the less affected hemisphere reflects a delayed but spatially similar progression to that of the initially affected hemisphere, this cannot be directly verified in the absence of presymptomatic imaging. Although this mirror-symmetric pattern has been documented in TDP-C and other neurodegenerative diseases such as Alzheimer’s disease, individual variability in disease spread or hemispheric vulnerability may limit the precision of this assumption. Finally, the demographic composition of the sample, which was predominantly White and non-Hispanic, may also limit the generalizability of the findings to more diverse populations.

Existing models of disease onset and propagation in TDP-C are limited. A defining feature of TDP-C pathology is the presence of long dystrophic neurites (DNs) in the superficial neocortical layers alongside a relative paucity of cortical neuronal cytoplasmic inclusions (NCIs) (Mackenzie et al., 2011). Post-mortem studies have demonstrated the presence of extensive cortical and subcortical TDP-C inclusions. However, post-mortem examination noted the burden of TDP-43 inclusions is often lower in areas with severe neuronal loss and gliosis, suggesting that inclusions disappear as affected neurons degenerate, complicating post-mortem disease staging (Kawles et al., 2022). The anterior temporal lobe’s prominence as an integrative hub for multimodal processing may make it disproportionately susceptible to disruptions caused by the accumulation of pathological TDP-43, particularly in neurons with long-range projections. However, further work is necessary to unravel the relationship between TDP-C and the anterior temporal lobe.

## Supplementary Material

Supplemental Figures

## Figures and Tables

**Table 1.** Demographic Characteristics of the TDP-C and Control (CON) Groups

	TDP-C	CON
N Visits A/B/C	40/28/13	64/0/0
Age at Visit A	63.0 (6.5)	63.5 (6.5)
% Female	47.6%	55.2%
% White, Non-Hispanic	96%	88%
CDR Global, Initial Visit	0.42 (0.3)	0.0 (0)
Age of Symptom Onset	58.5 (1.8)	
Autopsy	45%	

This table presents demographic and clinical information for participants in the TDP-C and Control (CON) groups. The TDP-C group includes 40, 28, and 13 participants at visits A, B, and C, respectively, while the CON group includes 64 participants at visit A. Age at Visit A is reported as mean (SD) for both groups. Additional data include the percentage of female participants, percentage of White, non-Hispanic participants, Clinical Dementia Rating (CDR) Global scores, age of symptom onset (for TDP-C only), and the percentage of participants with autopsy confirmation (for TDP-C only).

## Data Availability

The datasets and code supporting the findings of this study are available upon reasonable request from the corresponding author. Data sharing is subject to institutional and ethical guidelines.
