# Supplementary material for: Spatial and temporal progression of neurodegeneration in confirmed and suspected TDP-43 type C pathology
Source: Imaging Neurosci (Camb). 2025 Jul 16;3:IMAG.a.83. doi: 10.1162/IMAG.a.83 (PMC12320738; doi:10.1162/IMAG.a.83)
Supplement: Supplementary Material [file IMAG.a.83_supp.pdf]

Supplemental Figures

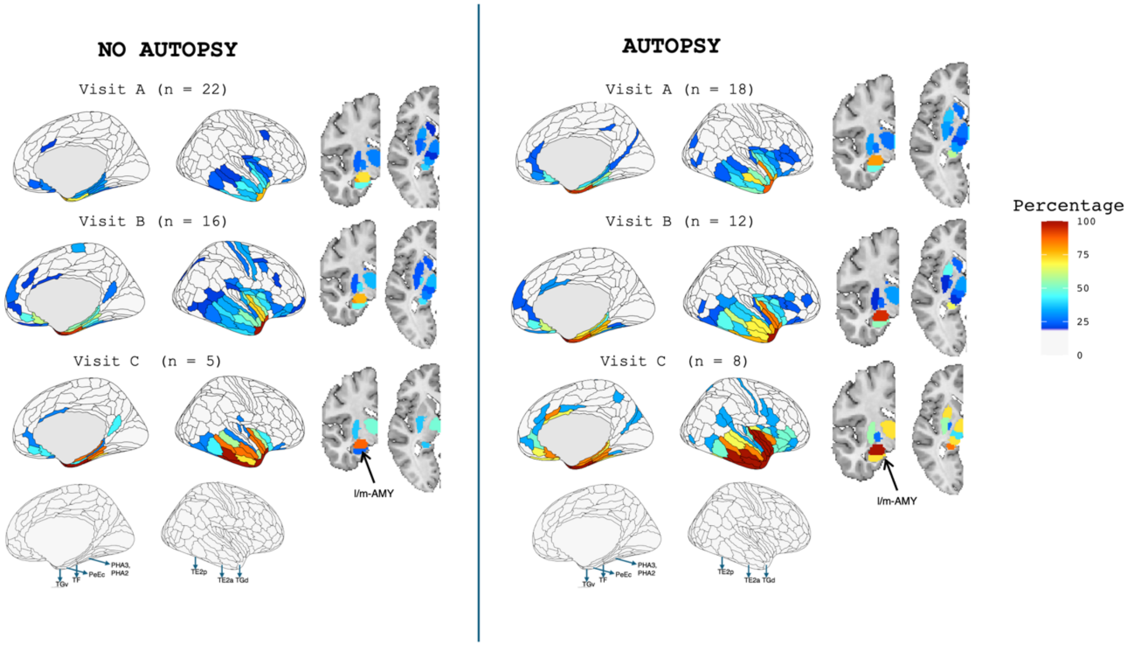

Supplemental Figure 1. Percentage of Participants with Brain Volume W-Scores Below -1.5 in Right Hemisphere ROIs at Visits A, B, and C

This figure illustrates the percentage of participants with brain volume W-scores below -1.5, based on comparison to normal controls, in 180 right hemisphere ROIs derived from the Glasser et al. (2018) HCP-MMP atlas for visits A, B, and C. Subcortical regions are parcellated according to the Tian et al. (2020) atlas. Left panel represents participants with suspected TDP-C while the right panel represents participants with TDP-C confirmed at autopsy. Visualizations are thresholded at 25%, displaying ROIs where 25% or more participants fall below the W-score threshold across the three visits. PRC = perirhinal cortex, EC = entorhinal cortex, aFG = anterior fusiform gyrus, PHA = parahippocampal area, PreS = presubiculum, pTG/VWFA = posterior temporal gyrus/visual word form area, midFG = middle fusiform gyrus, midMTG/ITG = middle and inferior temporal gyri, aMTG/ITG = anterior middle and inferior temporal gyri, pSTG/STS = posterior superior temporal gyrus and superior temporal sulcus, TPR = temporal pole region, Pir = piriform cortex, Ins = insula, and IFGorb = orbital portion of the inferior frontal gyrus. l/m-AMY = lateral and medial amygdala

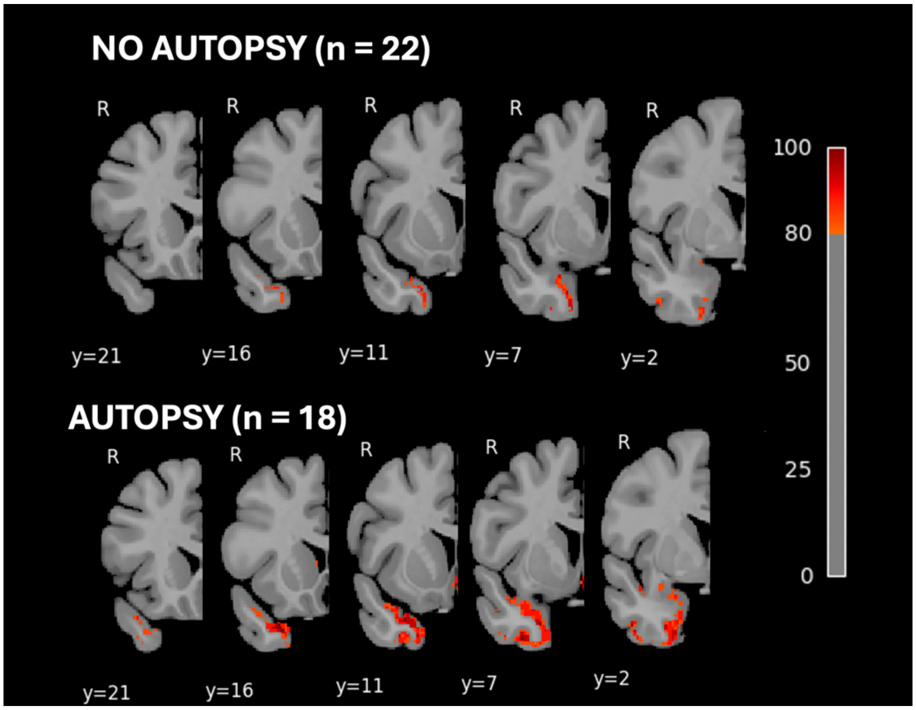

Supplemental Figure 2. Percentage of Participants with Brain Volume W-Scores Below -1.5 Visualized Voxelwise in the Right Hemisphere at Visit A

This figure presents voxelwise visualizations of the percentage of participants with brain volume W-scores below -1.5, in the right hemisphere, for visits A. Top row represents participants with suspected TDP-C while the bottom row represents participants with TDP-C confirmed at autopsy. Data are thresholded at 80%, with axial slices showing areas where 80% or more participants fall below the W-score threshold.

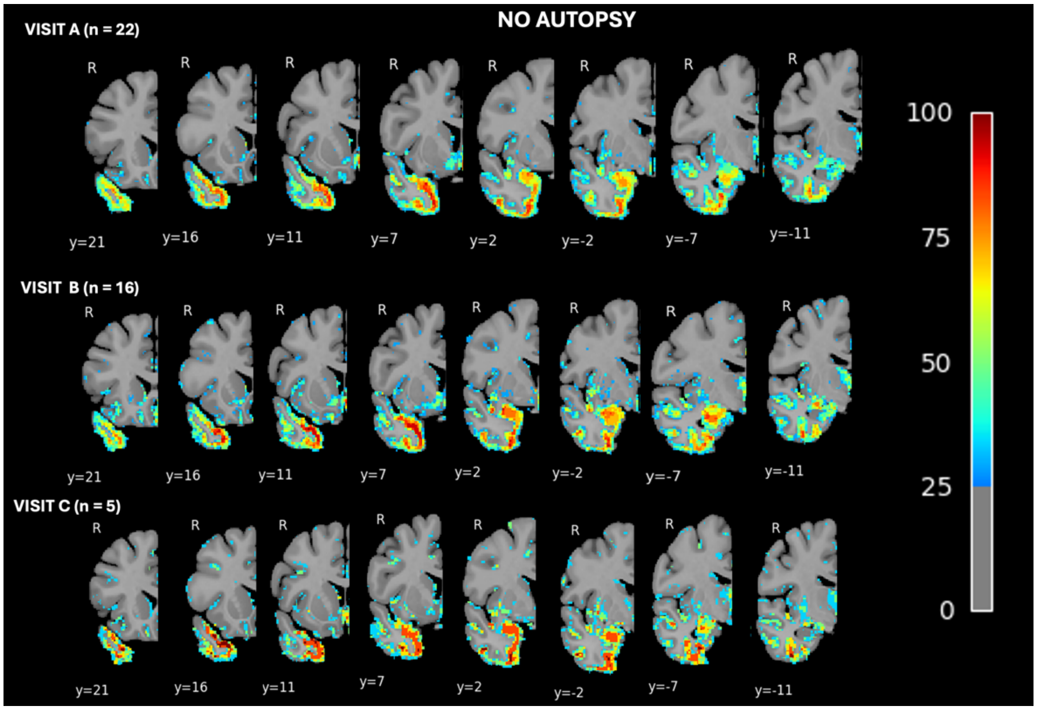

Supplemental Figure 3. Percentage of Participants with Brain Volume W-Scores Below -1.5 Visualized Voxelwise in the Right Hemisphere at Visits A, B, and C; No Autopsy Only

This figure presents voxelwise visualizations of the percentage of participants (suspected TDP-C only) with brain volume W-scores below -1.5, compared to normal controls, in the right hemisphere, for visits A, B, and C. Data are thresholded at 25%, with axial slices showing areas where 25% or more participants fall below the W-score threshold.

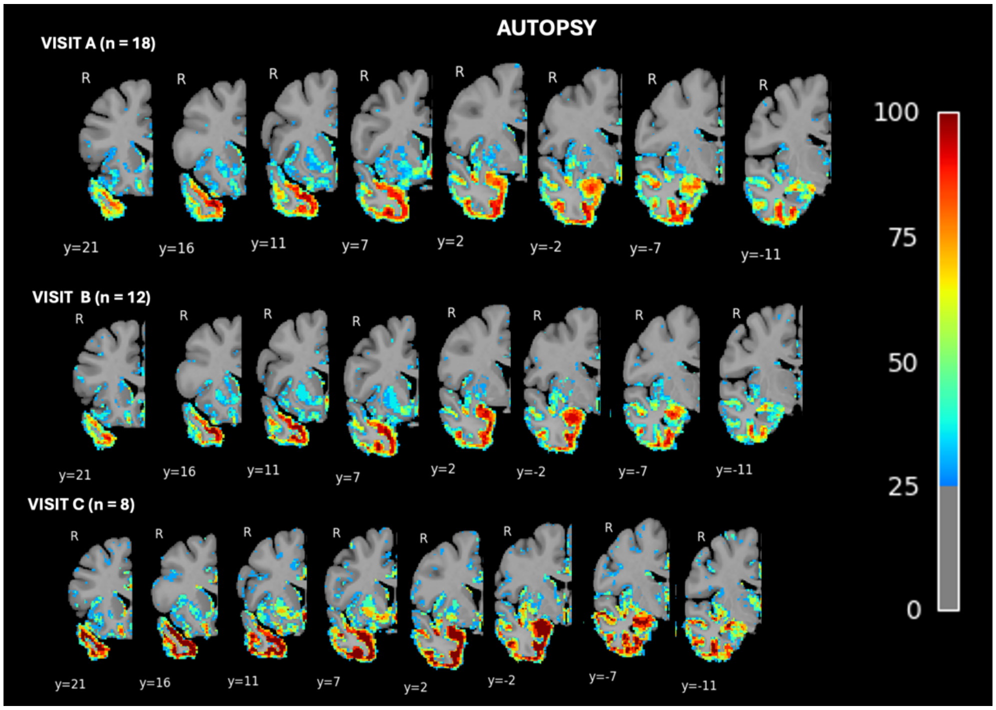

Supplemental Figure 4. Percentage of Participants with Brain Volume W-Scores Below -1.5 Visualized Voxelwise in the Right Hemisphere at Visits A, B, and C; Autopsy Only

This figure presents voxelwise visualizations of the percentage of participants (confirmed TDP-C) with brain volume W-scores below -1.5, compared to normal controls, in the right hemisphere, for visits A, B, and C. Data are thresholded at 25%, with axial slices showing areas where 25% or more participants fall below the W-score threshold.

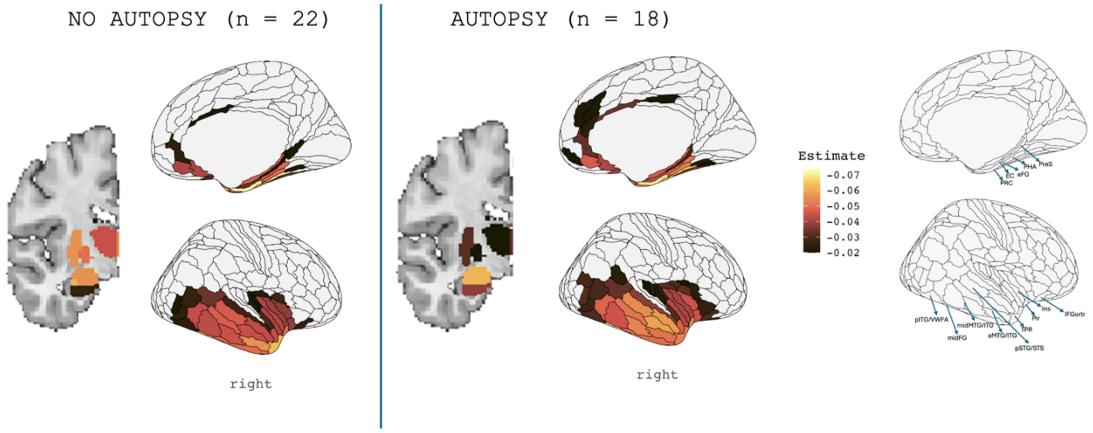

Supplemental Figure 5. Effects of Years Since Symptom Onset on Brain Volume by Autopsy

This figure displays the results of linear mixed-effects models examining the effect of disease duration on brain volume across 180 right-hemisphere regions of interest (ROIs) from the Glasser et al. (2018) HCP-MMP atlas and 16 subcortical ROIs from Tian et al. (2020). The left panel represents participants with suspected TDP-C while the right panel represents participants with TDP-C confirmed at autopsy. Lower estimate values (in lighter yellow) correspond to a faster rate of brain volume decline associated with the number of years since symptom onset. PRC = perirhinal cortex, EC = entorhinal cortex, aFG = anterior fusiform gyrus, PHA = parahippocampal area, PreS = presubiculum, pTG/VWFA = posterior temporal gyrus/visual word form area, midFG = middle fusiform gyrus, midMTG/ITG = middle and inferior temporal gyri, aMTG/ITG = anterior middle and inferior temporal gyri, pSTG/STS = posterior superior temporal gyrus and superior temporal sulcus, TPR = temporal pole region, Pir = piriform cortex, Ins = insula, and IFGorb = orbital portion of the inferior frontal gyrus.
